# The function of a conidia specific transcription factor CsgA in *Aspergillus nidulans*

**DOI:** 10.1038/s41598-022-19749-6

**Published:** 2022-09-16

**Authors:** He-Jin Cho, Hee-Soo Park

**Affiliations:** 1 School of Food Science and Biotechnology, Kyungpook National University, 80 Daehak-ro, Buk-gu, Daegu, 41566 Republic of Korea; 2 Department of Integrative Biology, Kyungpook National University, Daegu, 41566 Republic of Korea

**Keywords:** Microbiology, Fungi, Fungal genetics

## Abstract

*Aspergillus* spp. mainly reproduce asexually via asexual spores called conidia. In this study, we identified CsgA, a conidia-specific Zn_2_Cys_6_ transcription factor containing the GAL4-like zinc-finger domain, and characterized the roles of CsgA in the model organism *Aspergillus nidulans*. In *A. nidulans*, the Δ*csgA* strain produced abnormal conidiophores and exhibited increased conidial production. The deletion of *csgA* resulted in impaired production of sexual fruiting bodies (cleistothecia) and lower *mutA* expression levels. Overexpression of *csgA* led to decreased conidia production but increased cleistothecia production, suggesting that CsgA is essential for proper asexual and sexual development in *A. nidulans*. In conidia, the deletion of *csgA* resulted in increased trehalose content, higher spore viability, and increased tolerance to thermal and oxidative stresses. Transcriptomic analysis revealed that the loss of *csgA* affects the expression of genes related to conidia germination, DNA repair, and secondary metabolite biosynthesis. Further analysis revealed that the Δ*csgA* strain exhibited delayed conidial germination and abnormal germ tube length. Additionally, the production of sterigmatocystin increased in the Δ*csgA* conidia compared to that in the controls. Overall, these results suggest that CsgA is crucial for proper fungal development, spore viability, conidial germination, and sterigmatocystin production in *A. nidulans*.

## Introduction

*Aspergillus* species are filamentous fungi that are commonly found in soil, seeds, grains, and foods^1^. Some *Aspergillus* species can be used for food fermentation, enzyme production, and research purposes^2^; however, others act as opportunistic human pathogens or mycotoxin-producing fungi^3^. *Aspergillus nidulans* is an important model organism for filamentous fungi that is essential for genetic and fungal biology research but also produces a mycotoxin called sterigmatocystin^4^. Along with *A. nidulans*, several other *Aspergillus* species have been used to study biological processes in filamentous fungi^5^.

*A. nidulans* undergoes two reproductive modes: sexual and asexual. During sexual reproduction, *A. nidulans* produces sexual fruiting bodies called cleistothecia. However, *A. nidulans* primarily reproduces asexually via the production of conidia^6^. Conidia are formed from conidiophores, which are specialized asexual structures. During conidia formation and maturation, various morphological and physiological changes occur in the conidia, including changes to cell wall integrity, stress tolerance, and secondary metabolism required for conidial dormancy. These processes are complicated and various elements, especially transcription factors (TFs), play a vital role during conidiogenesis^6,7^.

TFs are proteins that have sequence-specific DNA-binding motifs and regulate the transcription of target genes^8^. Almost 80 TF families have been identified in the fungal genome^9^. In *A. nidulans*, TFs usually up- or downregulate gene expression during asexual development^5^. The initiation of conidiation is regulated by BrlA, a key TF in asexual development^10^. BrlA contains a C_2_H_2_ zinc finger DNA-binding domain^11^ and regulates the expression of AbaA, which controls the middle stage of conidiation^12^. AbaA, in turn, regulates the expression of WetA, a regulator of late-stage conidiation^13^. These three TFs have been defined as the central regulators of asexual development in *A. nidulans*^7^. Various TFs play crucial roles in asexual development following the initiation of conidiation by central regulators.

The zinc cluster family is the largest fungal-specific TF family^9^. Zinc cluster TFs have several DNA-binding motifs, such as the C_2_H_2_ zinc finger motif, C_4_ zinc finger motif, and C_6_ zinc finger motif^14^. The most studied zinc cluster protein is Gal4p (C_6_ zinc finger proteins), a transcriptional activator of genes involved in the catabolism of galactose in *Saccharomyces cerevisiae*^15^. The Gal4p superfamily plays various pivotal roles in fungal cells^15^. For example, AflR is a mycotoxin production-related TF that contains a C_6_ binuclear zinc cluster motif in *A. nidulans*^16^*.* In *A. flavus*, AflR not only regulates secondary metabolism, but also plays essential roles in fungal development^17^. SfgA, which encodes a GAL4-like Zn_2_Cys_6_ binuclear cluster DNA-binding domain, is one of the key negative regulators of conidiation in *A. nidulans* and *A. flavus*^18,19^. The Zn(II)_2_Cys_6_ transcription factor RosA acts as a repressor of sexual development in *A. nidulans*^20^. In addition, ZcfA includes a C_6_ binuclear zinc cluster motif that regulates asexual development, spore viability, and thermal stress tolerance in *A. nidulans* conidia^21^. Despite the large scale of the zinc cluster family, relatively little research has been conducted in *A. nidulans*.

In this study, we characterized a zinc cluster conidia-specific TF (*AN5955*) that encodes a GAL4-like Zn_2_Cys_6_ binuclear cluster DNA-binding domain. We designated *AN5955* as *csgA,* a conidia-specific GAL4-like zinc-finger protein, and elucidated the function of CsgA in *A. nidulans*.

## Results

### Summary of CsgA

**Figure 1:**
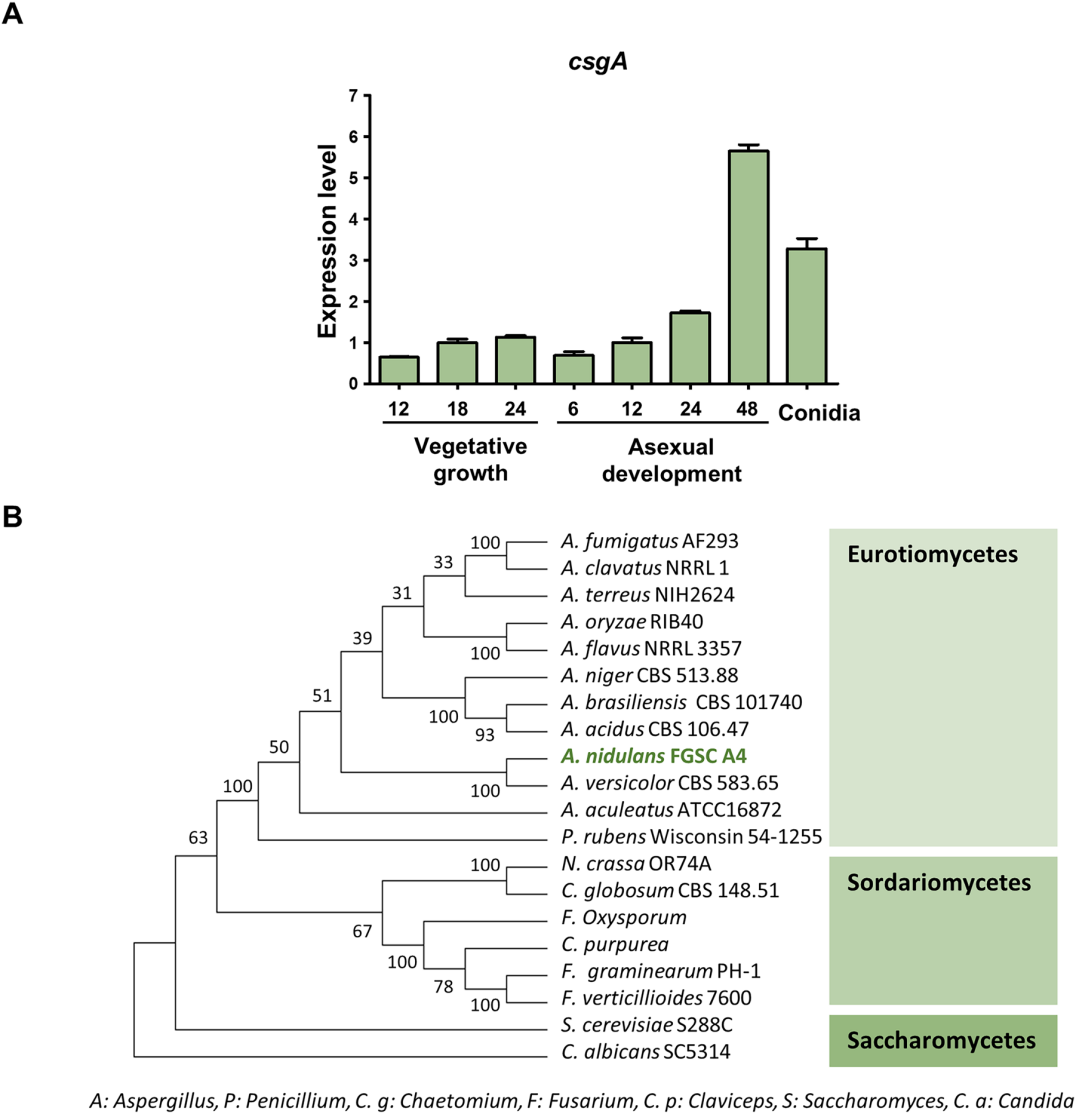
Summary of *csgA* evolution and expression. (**A**) Expression levels of *csgA* during the life cycle of *A. nidulans* measured by qRT-PCR. (**B**) Phylogenetic analysis of CsgA homolog proteins identified in ascomycota species, including *A. fumigatus* (XP_755373.1), *A. clavatus* (XP_001275339.1), *A. terreus* (XP_001213655.1), *A. oryzae* (XP_001826209.3), *A. flavus* (XP_041150305.1), *A. niger* (XP_001399754.2), *A. brasiliensis* (OJJ76189.1), *A. acidus* (OJZ92039.1), *A. nidulans* (XP_663559.1), *A. versicolor* (XP_040666950.1), *A. aculeatus* (XP_020060042.1), *Penicillium rubens* Wisconsin (XP_002565962.1), *Neurospora crassa* (KHE82752.1), *Chaetomium globosum* (XP_001223925.1), *Fusarium oxysporum* (EXA29503.1), *Claviceps purpurea* (KAG6216579.1), *F. graminearum* (XP_011323014.1), *F. verticillioides* (XP_018761247.1), *Saccharomyces cerevisiae* (NP_013199.1), and *Candida albicans* (XP_019330616.1).

Previously, we screened mRNA expression of gene encoding zinc cluster transcription factors which contain a GAL4-like Zn_2_Cys_6_ binuclear cluster DNA-binding domain in hyphae and conidia, and found that mRNA levels of 8 genes were highly expressed in conidia compared to hyphae (Fig. S1). We then generated deletion mutants for each gene and checked colony morphology. Based on phenotype analysis, deletion of *AN5955* affected growth and development. Therefore, we hypothesized that *AN5955* plays important role in *A. nidulans* conidia and named *AN5955* as conidia-specific GAL4-like zinc finger protein (*csgA*). *AN5955* encodes a protein containing a GAL4-like Zn_2_Cys_6_ binuclear cluster DNA-binding domain; therefore, *AN5955* was named conidia-specific GAL4-like zinc finger protein (*csgA*). We then checked the mRNA expression levels of *csgA* during the life cycle of *A. nidulans*, which were measured using real-time quantitative reverse transcription-polymerase chain reaction (qRT-PCR) (Fig. 1A). The expression levels of *csgA* increased during the late stage of asexual development and were high in conidia. To identify homologs that contain the same domain in various ascomycota species, FungiDB (http://www.fungiDB.org)^22^ and NCBI blastp were used to search *Aspergillus* and non-*Aspergillus* species, respectively. Based on the genome database, a phylogenetic tree was generated using MEGA 5 software (http://www.megasoftware.net) using alignment data from ClustalW2 (Fig. 1B). CsgA is conserved in various Ascomycota classes, including Eurotiomycetes, Sordariomycetes, and Saccharomycetes.

### CsgA is involved in asexual development

**Figure 2:**
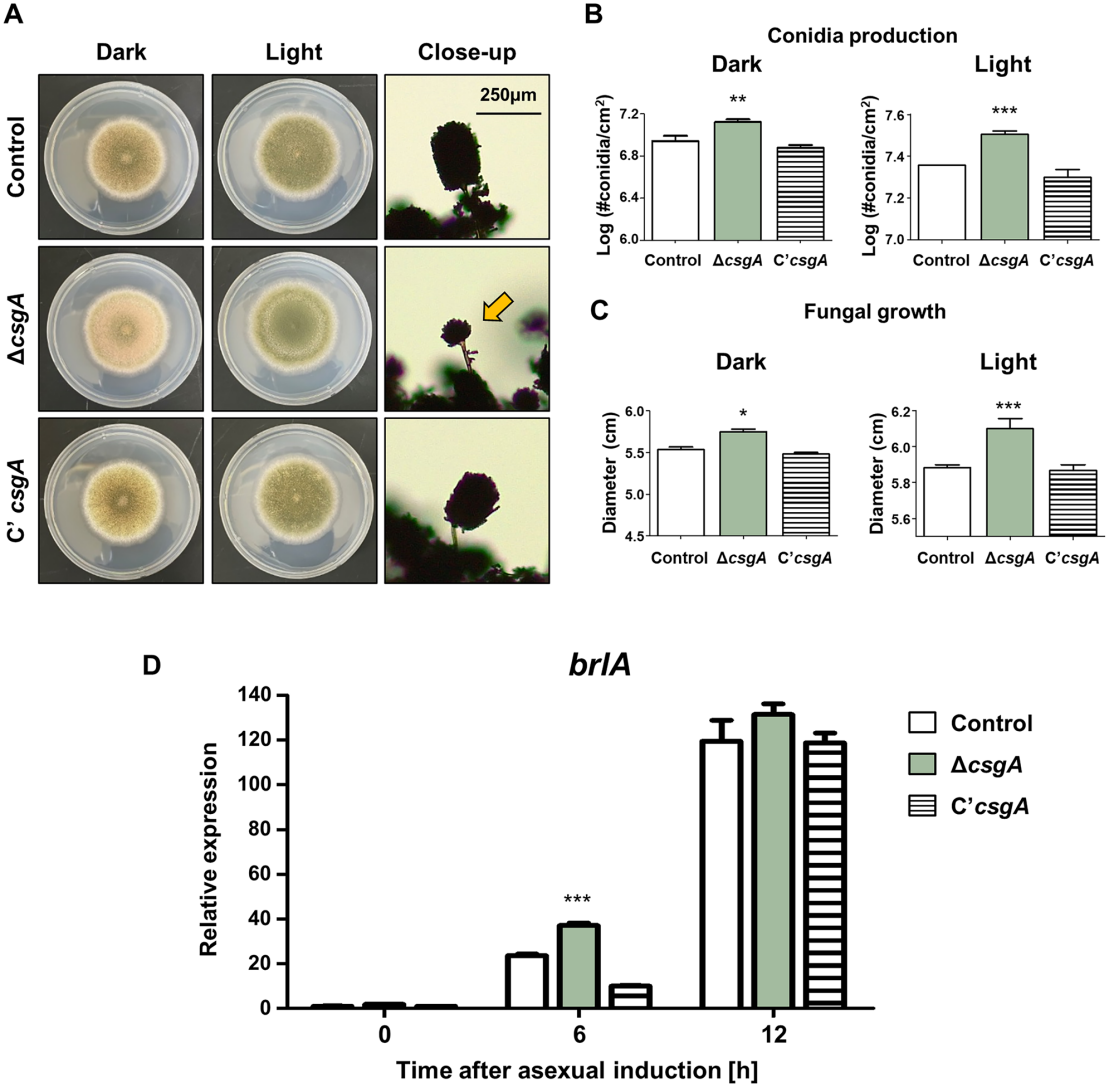
The roles of *csgA* in asexual development. (**A**) Colony photographs of control (TNJ36), Δ*csgA* (THJ13.1), and C'*csgA* (THJ28.1) strains point-inoculated on solid MMG, grown for 5 days at 37 °C in dark and light conditions. Close-ups show conidiophores of each strain. (**B**) Quantitative analyses of conidia production by each strain shown in (**A**). (**C**) Quantitative analyses of fungal growth of each strain. *p < 0.05, **p < 0.01, ***p < 0.001. (**D**) The mRNA expression of *brlA* in conidia. qRT-PCR analyses of the mRNA expression levels of *brlA* in each strain after asexual induction. (***p < 0.001).

To functionally characterize *csgA*, deletion mutant (Δ*csgA*) and complemented strains (C’*csgA*) were generated. Control, Δ*csgA,* and C’*csgA* strains were point inoculated onto a solid minimal medium with 1% glucose (MMG) and incubated at 37 °C for 5 days under dark and light conditions (Fig. 2A). The conidiophores of the Δ*csgA* strain were smaller than those of the control and C’*csgA* strains. Compared to the control strain, the Δ*csgA* strain produced more conidia per unit area under both dark and light conditions (Fig. 2B). As shown in Fig. 2C, the Δ*csgA* strain exhibited higher colony diameter under both dark and light conditions. In addition, deletion of *csgA* led to increase in *brlA* expression in the early stages of conidiation (Fig. 2D). These results indicate that CsgA is essential for the proper asexual development of *A. nidulans*.

### CsgA plays a role in sexual development

**Figure 3:**
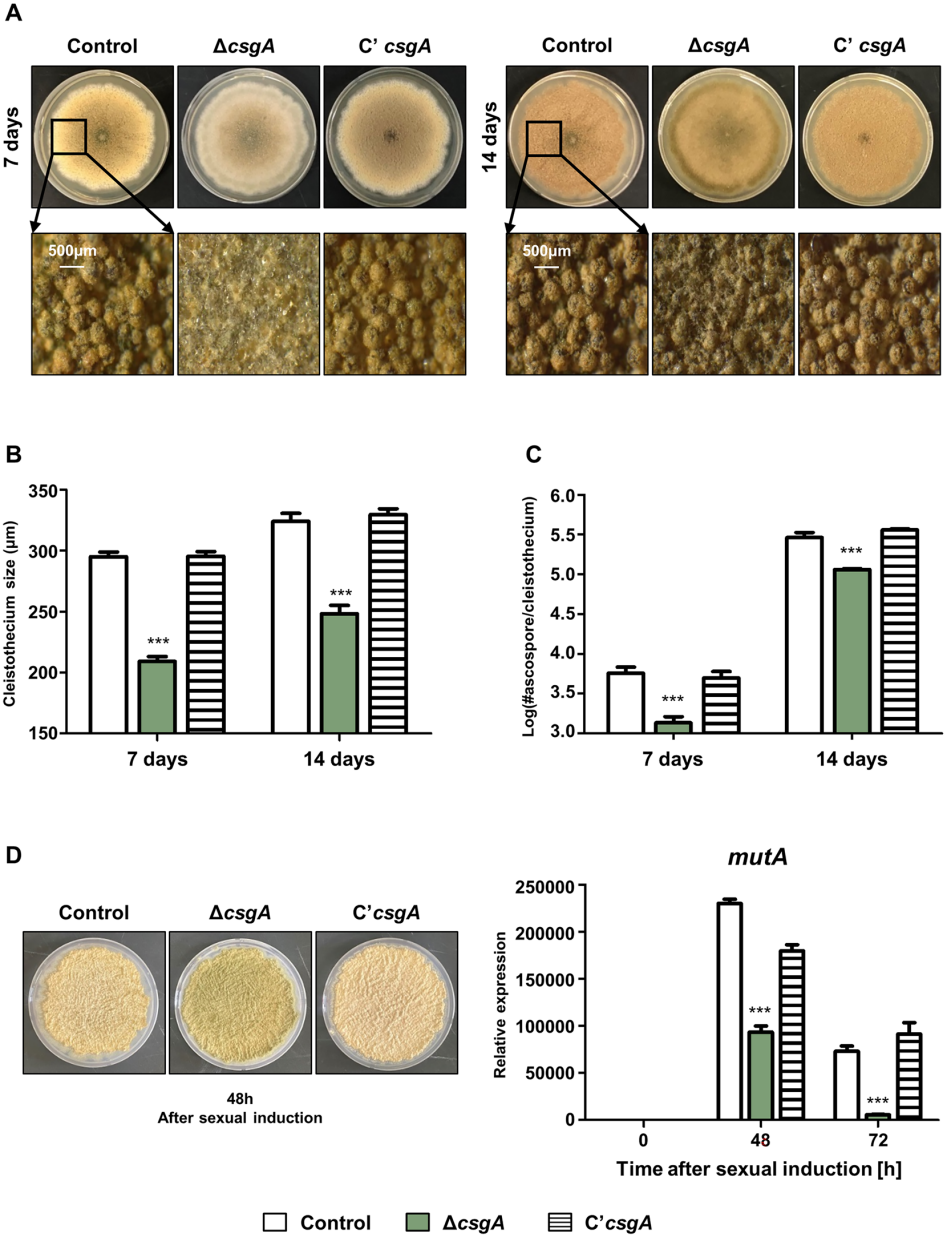
The roles of *csgA* in sexual development. (**A**) Colony photographs of control (TNJ36), Δ*csgA* (THJ13.1), and C'*csgA* (THJ28.1) strains point-inoculated on solid SM and grown for 7 days and 14 days at 37 °C in dark condition. (**B**) Sizes of a single cleistothecium from each strain shown in (**A**). (**C**) Germination of ascospores from each strain. (**D**) Left, colony photographs of each strain at 48 h after sexual induction. Right, qRT-PCR analyses of *mutA* expression in each strain. ***p < 0.001.

To investigate the function of CsgA in sexual development, control, Δ*csgA,* and C’*csgA* strains were point inoculated onto a solid sexual medium (SM) and incubated at 37 °C for 7 days and 14 days (Fig. 3A). First, we collected cleistothecia from the media and measured the size of the cleistothecium. Cleistothecium size of the Δ*csgA* strain was smaller than that of the control and C’*csgA* strains at 7 days (Fig. 3B). The size of the Δ*csgA* cleistothecium after 14 days was larger than that of the Δ*csgA* cleistothecium after 7 days, but was still smaller than that of the control strain (Fig. 3B). To examine the germination ability of sexual spores (ascospores) from control, Δ*csgA,* and C’*csgA* strains, 100 ascospores from each strain were spread onto solid MMG and incubated at 37 °C for 2 days. After incubation, the colonies were counted and the number of ascospores from a single cleistothecium was calculated. As shown in Fig. 3C, the germination ability of Δ*csgA* ascospores was significantly lower than that of the control or C’*csgA* strains at 7 and 14 days. To further characterize the roles of CsgA in sexual development, we assessed the mRNA expression levels of *mutA*, a gene that is highly expressed during sexual development^23^. The Δ*csgA* strain exhibited reduced mRNA expression levels of *mutA* compared to the control strain (Fig. 3D). These results indicate that CsgA plays a crucial role in the sexual development of *A. nidulans*.

### *CsgA* is essential for balancing sexual and asexual development

**Figure 4:**
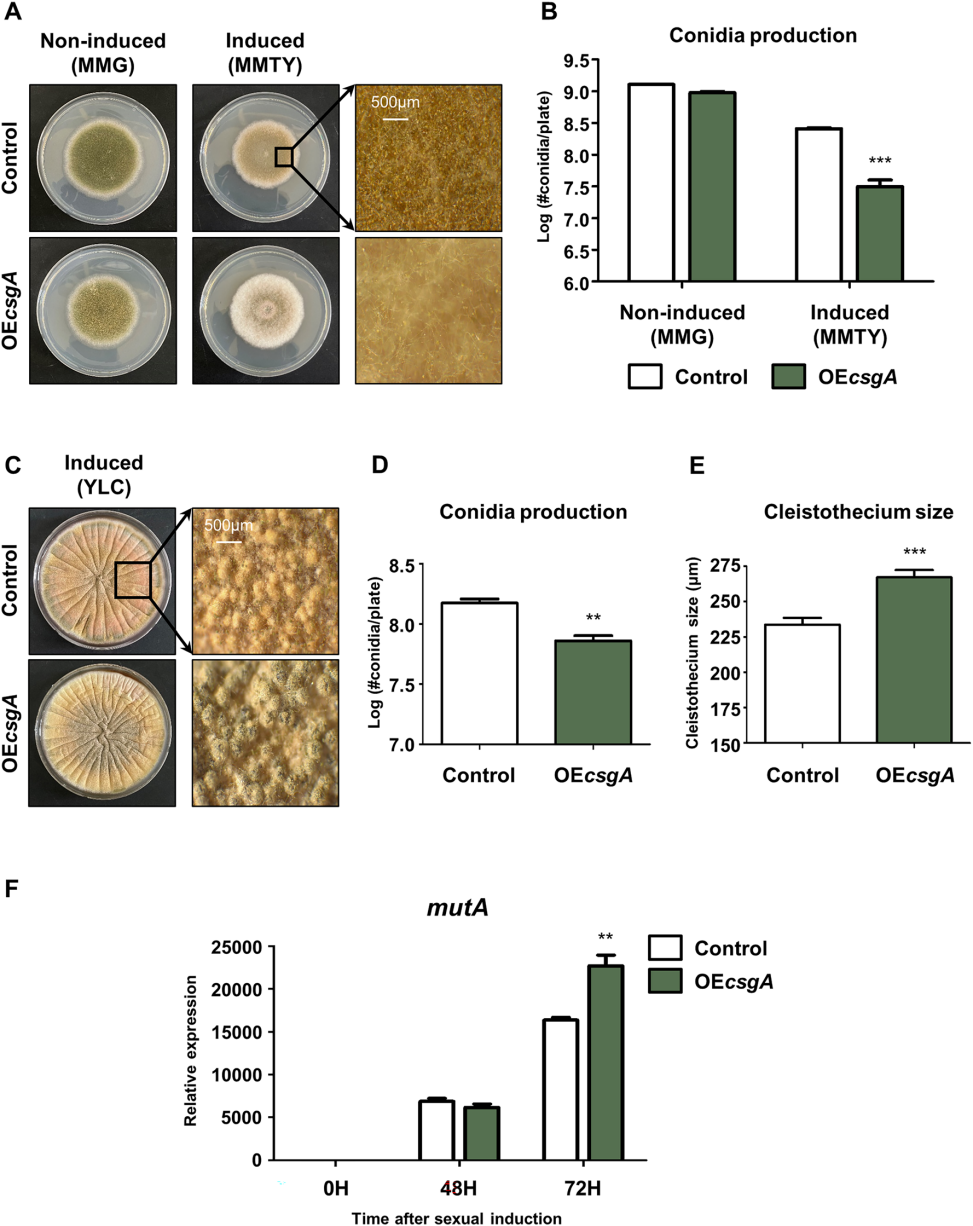
Effect of *csgA* overexpression. (**A**) Colony photographs of control (THS30) and OE*csgA* (THJ27.1) strains point-inoculated on solid MMG and MMTY and grown for 5 days at 37 °C in light conditions. (**B**) Quantitative analyses of conidia production in each strain shown in (**A**). (**C**) Colony photographs of control (THS30) and OE*csgA* (THJ27.1) strains point-inoculated on solid YLC and grown for 5 days at 37 °C in light conditions. (**D**) Quantitative analyses of conidia production of each strain shown in (**C**). (**E**) Quantitative analyses of sexual development in each strain by measuring single cleistothecium size. (**F**) mRNA expression levels of *mutA* in each strain grown on solid YLC after sexual development induction. **p < 0.01, ***p < 0.001.

To examine the role of CsgA in fungal development, a *csgA* overexpression strain (OE*csgA*) was generated. Control and OE*csgA* strains were point inoculated onto a solid MMG (non-inducing medium) or minimal medium with 100 mM threonine as carbon source and 0.1% yeast extract (MMTY, inducing medium) at 37 °C for 5 days under light conditions (Fig. 4A). When inoculated on MMTY, the OE*csgA* strain exhibited lower conidial production compared to the control strain (Fig. 4B). To further investigate the functions of *csgA* overexpression in fungal development, control and OE*csgA* strains were point-inoculated on solid yeast extract-lactose-cyclopentanone (YLC, inducing medium) (Fig. 4C). Again, conidia production in the OEc*sgA* strain was lower than that in the control strain (Fig. 4D). Next, sexual development was assessed by measuring cleistothecium size. As shown in Fig. 4E, the OE*csgA* strain exhibited enhanced sexual development compared to the control strain. Additionally, the OE*csgA* strain exhibited increased mRNA expression levels of *mutA* compared to the control strain (Fig. 4F). As expected, the *csgA* overexpression phenotype (reduced asexual development and increased sexual development) was opposite to that observed in the Δ*csgA* strain. Overall, these results indicate that CsgA is essential for maintaining the balance between asexual and sexual development in *A. nidulans.*

### CsgA is involved in trehalose biosynthesis and stress tolerance

**Figure 5:**
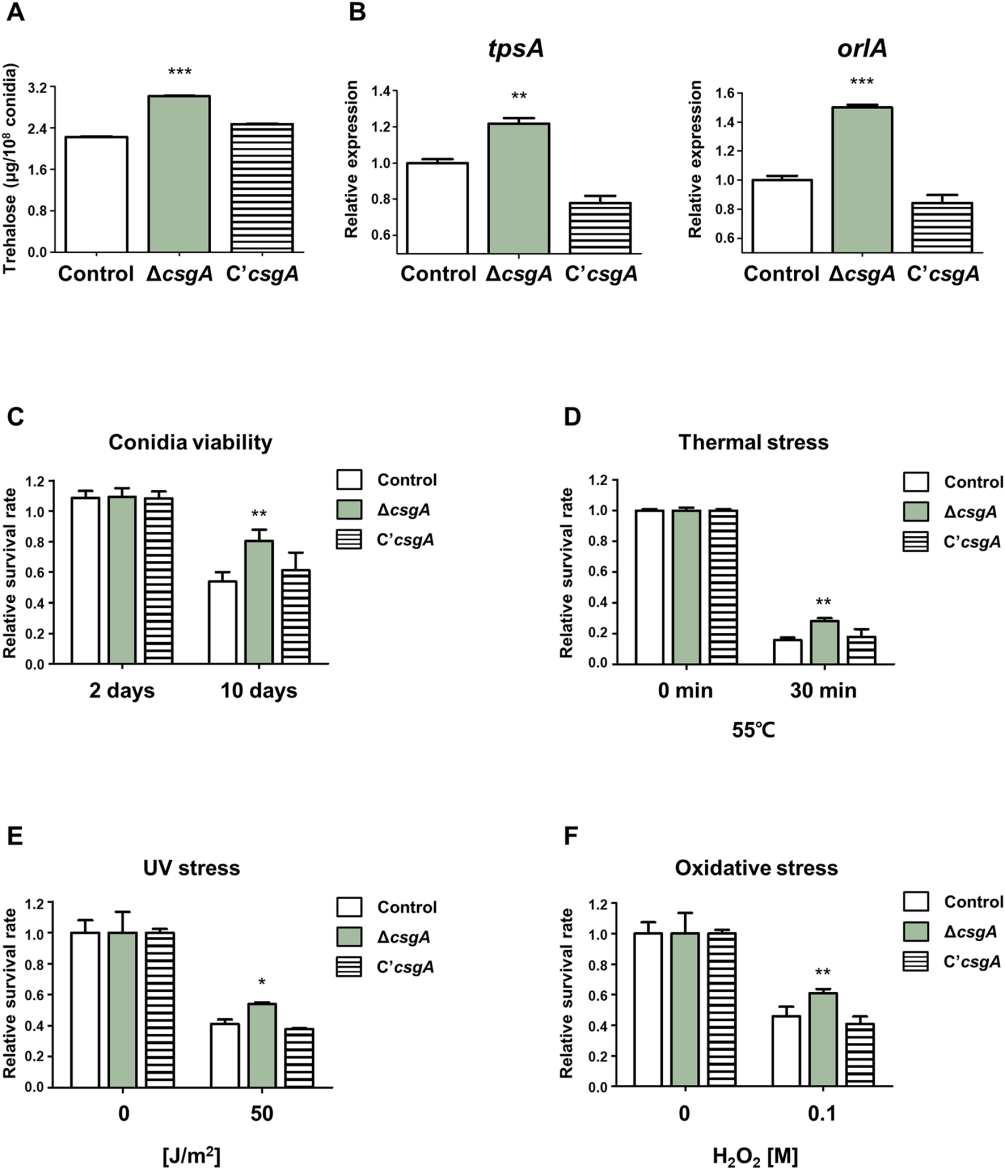
Characterization of *csgA* function in conidia. (**A**) Trehalose levels in conidia from control (TNJ36), Δ*csgA* (THJ13.1), and C'*csgA* (THJ28.1) strains. (**B**) mRNA expression levels of *tpsA* and *orlA* in conidia from each strain. (**C**) Viability of conidia from each strain grown for 2 and 10 days. (**D**) Thermal stress response of each strain following exposure to 55 °C temperature. (**E**) UV stress response of each strain following exposure to UV light. (**F**) Oxidative stress response of each strain following exposure to 0.1 M H_2_O_2_. *p < 0.05, **p < 0.01, ***p < 0.001.

To investigate the function of CsgA in conidia, the amount of trehalose, a stress protectant, and stress tolerance were measured. Total trehalose content was higher in the Δ*csgA* strain than that in the control strain (Fig. 5A). In addition, the mRNA expression levels of genes related to trehalose biosynthesis, including *tpsA*^24^ and *orlA*^25^, were higher in the Δ*csgA* strain (Fig. 5B). Because trehalose acts as a stress protectant^26^, we proceeded to examine conidial viability and stress tolerance in conidia. As shown in Fig. 5C, conidial viability in Δ*csgA* conidia was higher than that in the control. Stress tolerance tests against thermal, UV, and oxidative stresses were performed, and we found that the Δ*csgA* strain was more resistant to high-temperature, UV, and oxidative stresses compared to the control strain (Fig. 5D–F). Taken together, these results indicate that CsgA is involved in trehalose biosynthesis, conidial viability, and stress tolerance in *A. nidulans* conidia.

### Transcriptomic analysis of Δ*csgA* conidia

**Figure 6:**
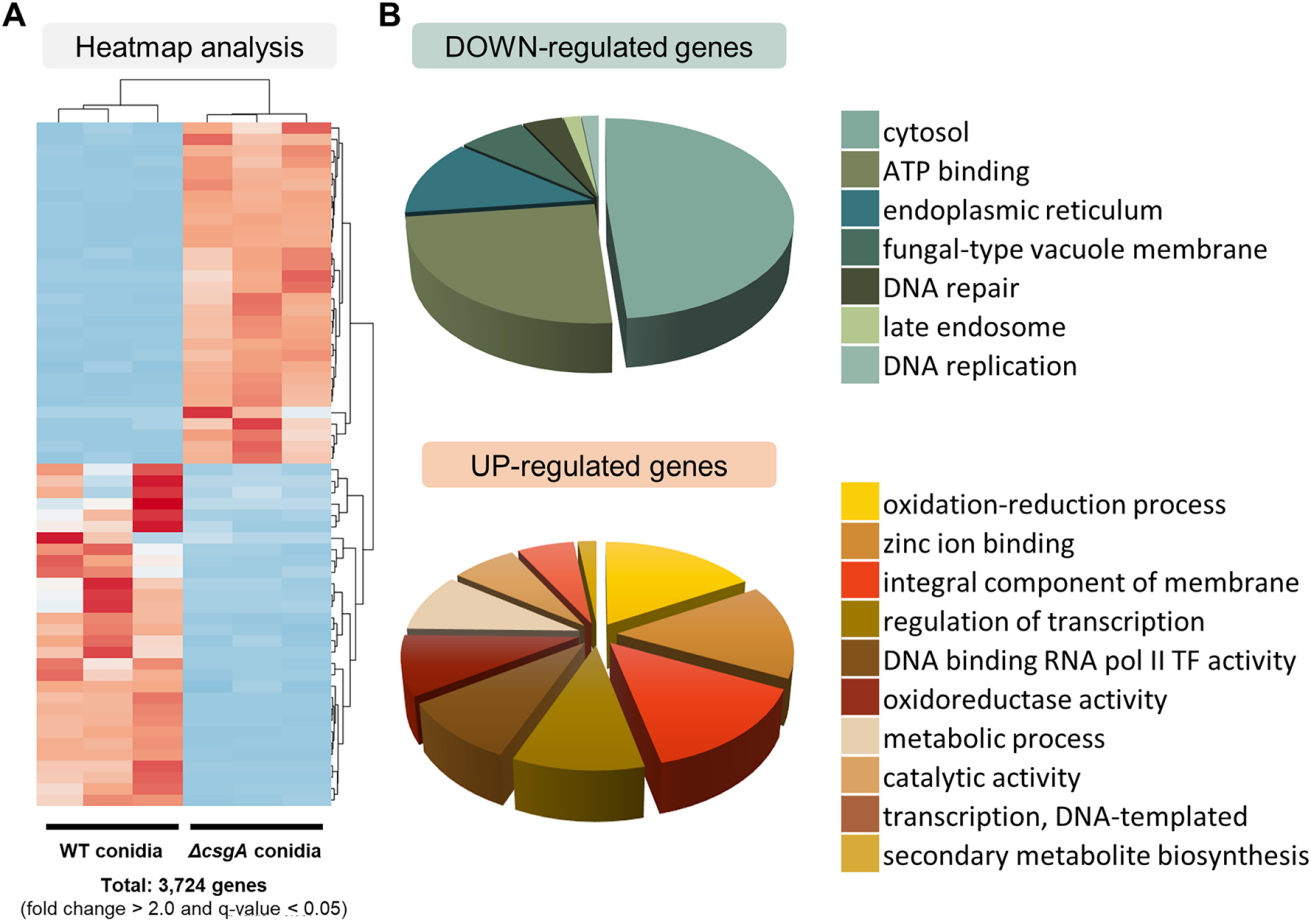
Transcriptomic analysis of Δ*csgA* conidia. (**A**) Heat map of differentially expressed genes (DEGs) between WT and Δ*csgA* conidia (fold change > 2.0; q-value < 0.05). (**B**) GO term enrichment analyses of down-regulated and up-regulated genes in Δ*csgA* conidia.

As mentioned above, CsgA is essential for proper conidia formation, maturation, and stress tolerance in *A. nidulans*. To further elucidate the role of CsgA in conidia, RNA-sequencing (RNA-seq) of conidia from two-day-old wild-type (WT) and Δ*csgA* strains was performed. We detected 3724 differentially expressed genes (DEGs) between the strains (fold change > 2.0; q-value < 0.05) (Fig. 6A). Among them, 1879 genes were upregulated and 1845 genes were downregulated in the Δ*csgA* conidia compared to that in the WT conidia. To further elucidate the role of CsgA, gene ontology (GO) term enrichment analyses were performed using the FungiFun platform^27^ (Fig. 6B). Genes associated with the cytosol, ATP binding, and fungal-type vacuole membrane were downregulated in the Δ*csgA* conidia, whereas genes related to secondary metabolite biosynthetic processes, metabolic processes, zinc ion binding, regulation of transcription, and catalytic activity were upregulated in the Δ*csgA* conidia, implying that CsgA regulates genes associated with metabolism, thereby controlling primary or secondary metabolism in conidia.

### CsgA regulates germination in *A. nidulans* conidia

**Figure 7:**
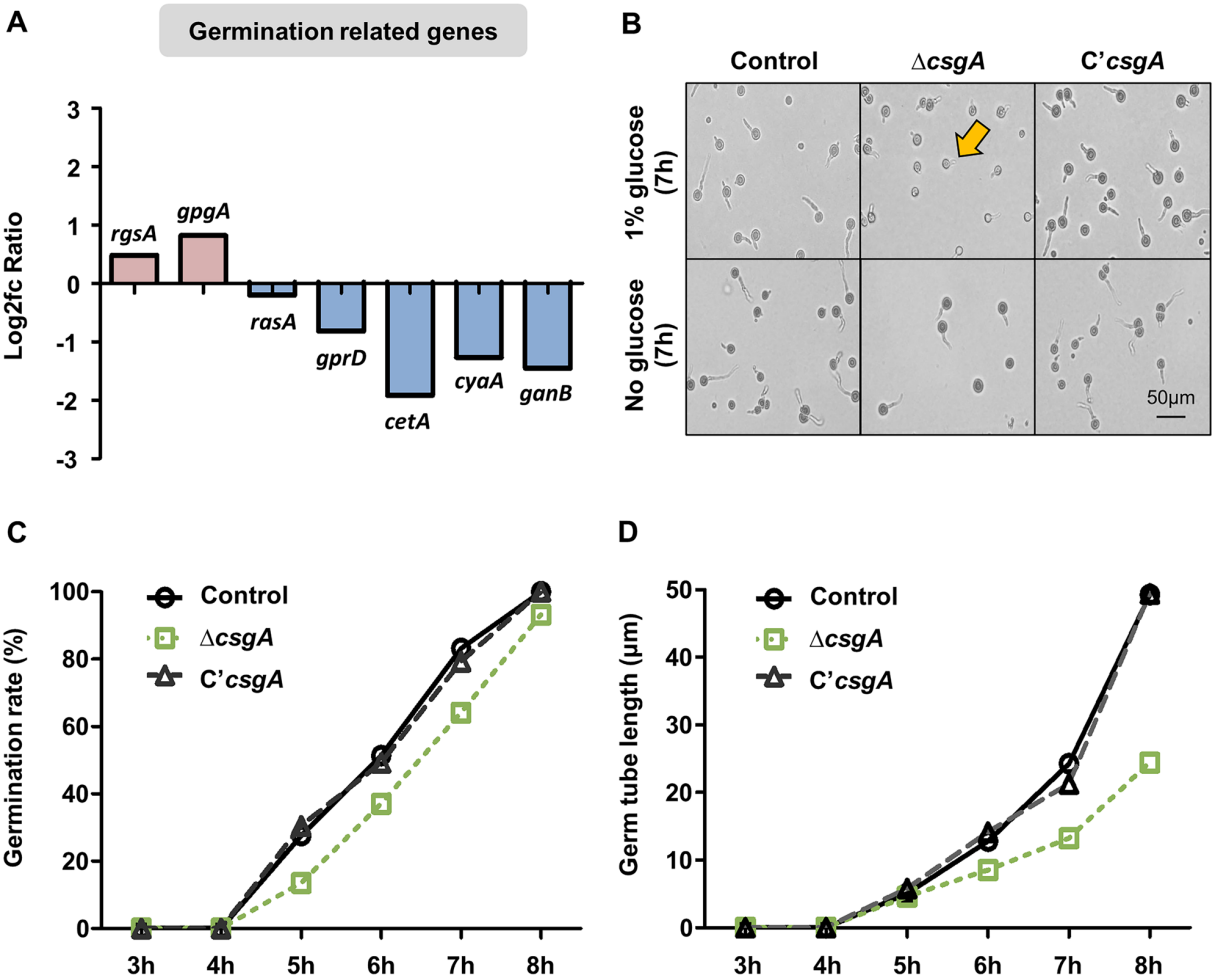
The role of *csgA* in conidial germination. (**A**) mRNA expression levels of genes involved in conidial germination in Δ*csgA* conidia. (**B**) Colony photographs of control (TNJ36), Δ*csgA* (THJ13.1), and C'*csgA* (THJ28.1) strains inoculated on solid MM with or without 1% glucose and incubated for 8 h. (**C**) Germination rate of each strain assessed hourly until germination reached 100%. (**D**) Germ tube length of each strain assessed hourly.

Our transcriptomic analysis revealed that the deletion of *csgA* affected the mRNA expression levels of genes related to spore germination, including *rgsA*^28^, *gpgA*^29^, *rasA*^30^, *grpD*^31^, *cetA*^32^, *cyaA*^33^, and *ganB*^34^ (Fig. 7A). mRNA levels of these genes were verified using reverse-transcription qPCR analysis (Fig. S2). Based on this finding, we proceeded to measure the germination rate over time in the conidia of the control, Δ*csgA*, and C'*csgA* strains (Fig. 7B). The Δ*csgA* conidia exhibited a slightly delayed germination rate compared to that of the control and C’*csgA* strains, which appeared to catch up by 8 h (Fig. 7C). The germ tube length was shorter in the Δ*csgA* strain compared to that in the control and C’*csgA* strains (Fig. 7D). These results indicate that CsgA is required for proper conidial germination in *A. nidulans*.

### CsgA is involved in sterigmatocystin production

**Figure 8:**
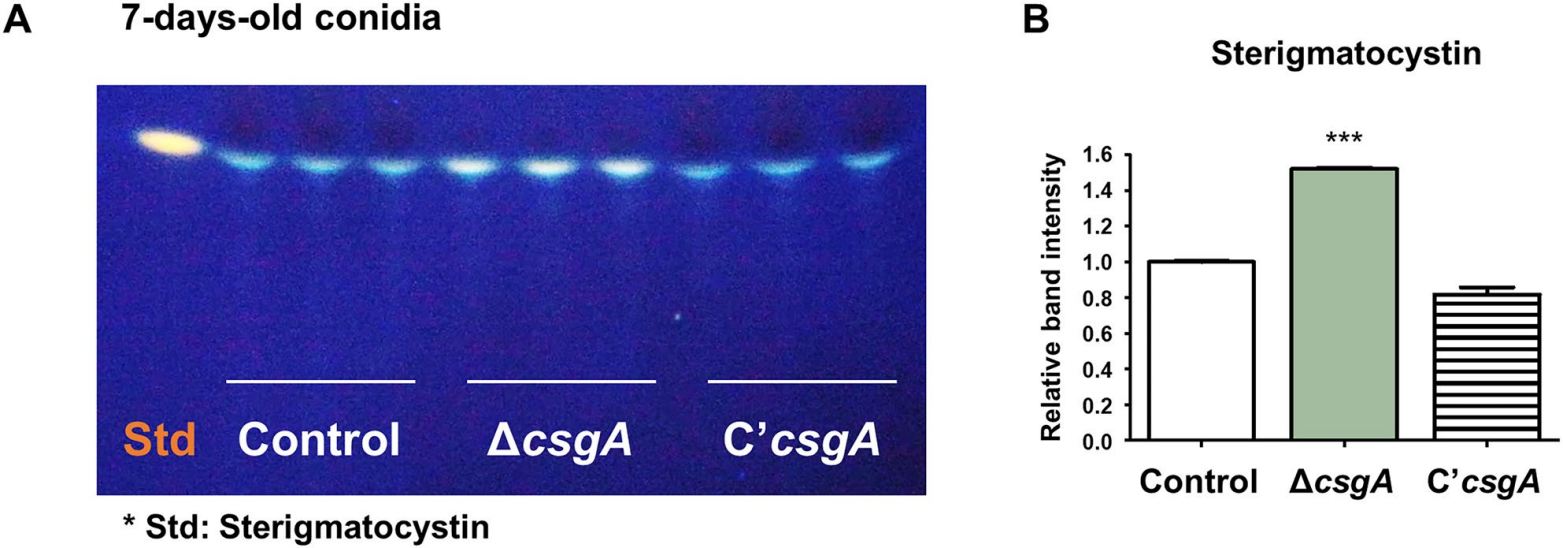
Function of *csgA* in sterigmatocystin production. (**A**) TLC plate showing the production of sterigmatocystin by 7-day-old conidia from control (TNJ36), Δ*csgA* (THJ13.1), and C'*csgA* (THJ28.1) strains. (**B**) Quantitative analyses of band intensities from each strain using ImageJ software. ***p < 0.001.

To investigate the role of CsgA in sterigmatocystin production, the amount of sterigmatocystin was quantified in control, Δ*csgA,* and C’*csgA* strains. Sterigmatocystin was extracted from each strain and spotted on thin-layer chromatography (TLC) plates. Compared to that of the control strain, conidia from the Δ*csgA* strain produced significantly more sterigmatocystin (Fig. 8A). As shown in Fig. 8B, the relative band intensities on the TLC plates were analyzed, and the Δ*csgA* strain displayed higher band intensities than the control strain. These results demonstrate that CsgA is essential for proper production of sterigmatocystin in *A. nidulans* conidia.

### The roles of CsgA in *A. nidulans* ascospores

**Figure 9:**
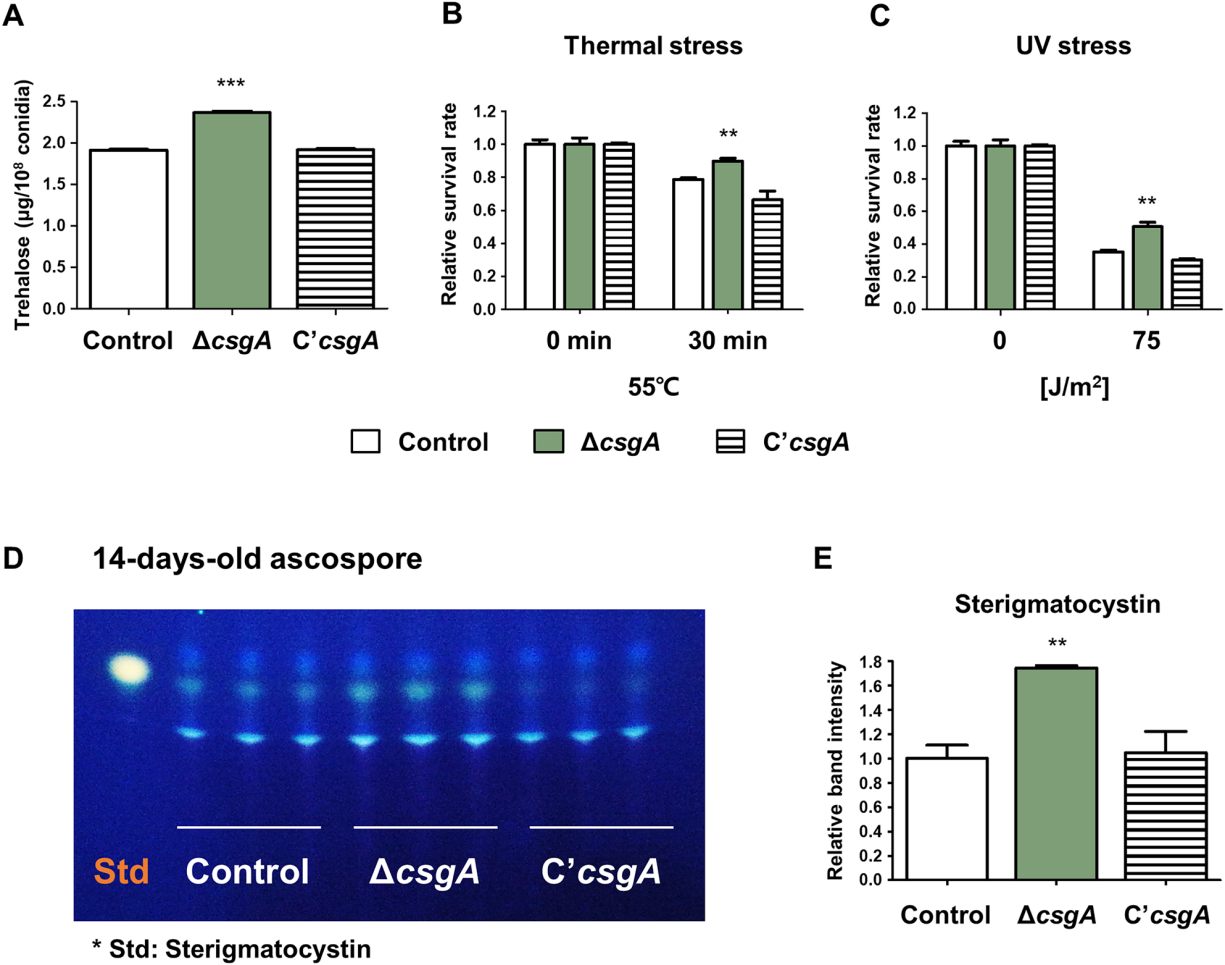
The roles of *csgA* in ascospores. (**A**) Trehalose levels in ascospores from control (TNJ36), Δ*csgA* (THJ13.1), and C'*csgA* (THJ28.1) strains. (**B**) Thermal stress response of each strain following exposure to 55 °C. (**C**) UV stress response of each strain following exposure to UV light. (**D**) TLC plate showing the production of sterigmatocystin from 14-day-old ascospores from control (TNJ36), Δ*csgA* (THJ13.1), and C'*csgA* (THJ28.1) strains. (**E**) Quantitative analyses of band intensities of each strain using ImageJ software. **p < 0.01, ***p < 0.001.

Because CsgA affects trehalose biosynthesis, stress tolerance, and sterigmatocystin production in asexual spores, we hypothesized that CsgA may play a similar role in ascospores. To investigate the function of CsgA in *A. nidulans* ascospores, trehalose content and stress tolerance were measured. Total trehalose content was higher in the Δ*csgA* strain than that in the control strain (Fig. 9A). The Δ*csgA* strain exhibited higher stress tolerance under thermal and UV stresses compared to the control strain (Fig. 9B,C). We also examined sterigmatocystin production in ascospores and found that the amount of sterigmatocystin in Δ*csgA* ascospores was higher than that in control and C’*csgA* ascospores (Fig. 9D,E). Overall, these results indicate that CsgA plays similar roles in the asexual and sexual spores of *A. nidulans*.

## Discussion

*Aspergillus* species mainly reproduce through asexual reproduction, and many transcription factors are required for this process^5^. Among them, BrlA, AbaA, and WetA have been defined as the central regulators of gene expression related to conidiation^6,35^. There is a wide variety of TF families in the fungal genome, the largest of which is the fungal-specific zinc cluster family^9^. Zinc cluster TFs are known to be involved in various biological processes in fungal cells^15^. The functions of zinc cluster TFs have been well studied in *Saccharomyces cerevisiae*, and the most studied zinc cluster protein is Gal4p (C_6_ zinc finger protein), which plays a variety of pivotal roles in fungal cells^15^. In *A. nidulans*, only a few GAL4-like proteins have been studied. For example, McrA, which encodes a GAL4-like Zn_2_Cys_6_ domain at the C-terminus, plays a key role in fungal growth, spore viability, and secondary metabolism^36^. ZcfA, a zinc cluster TF containing a GAL4-like Zn_2_Cys_6_ binuclear cluster DNA-binding domain, also regulates both asexual and sexual development in *A. nidulans*^21^. In addition, VadZ, which contains a GAL4-type Zn_2_Cys_6_ binuclear cluster DNA-binding domain, regulates asexual and sexual development and sterigmatocystin production^37^. In this study, we studied the roles of the conidia-specific TF CsgA, which is a GAL4 type TF, in fungal development and mycotoxin production in *A. nidulans*.

We characterized the role of CsgA in fungal development. The Δ*csgA* strain showed increased fungal growth and conidiation, but the conidiophore size was smaller than that of the control and C’*csgA* strains. The expression level of *brlA* in the Δ*csgA* strain increased during the early stages of conidiation (Fig. 2), suggesting that CsgA may regulate the early stages of the conidiation process. In addition, sexual development of the Δ*csgA* strain was abnormal; the sexual fruiting structure size of the Δ*csgA* strain was smaller than that of the control strain, regardless of incubation time. Overexpression of *csgA* resulted in decreased conidial production and increased sexual development (Fig. 3), suggesting that CsgA is essential for maintaining a balance between asexual and sexual development in *A. nidulans*. Although the role of CsgA was studied in asexual development based on phenotypic analysis, the genetic relationship between CsgA and othe developmental transcription factors has not yet been studied in depth. To check the relationship between CsgA and BrlA/AbaA, we checked the *brlA* response element (BRE) and the *abaA* response element (ARE) in the *csgA* promoter region. However, BRE and ARE were not found in the *csgA* promoter region, implying that BrlA or AbaA may not directly regulate *csgA* expression during asexual development. Further research how to control mRNA expression of *csgA* during asexual development will be needed to understand the genetic network in asexual development. Also, the relationship between CsgA and the upstream developmental activators such as FlbA–E, should be studied in initiation of asxual development.

As CsgA is a conidia-specific TF, its function in *A. nidulans* conidia was investigated. Trehalose content increased in the Δ*csgA* strain compared to that in the control and C’*csgA* strains. Because trehalose is related to stress tolerance and spore viability^26^, the Δ*csgA* strain also showed higher viability and tolerance to various stresses. These results indicate that CsgA plays a crucial role in conidial viability in *A. nidulans*. Interestingly, transcriptomic analyses revealed no significant changes in the mRNA expression levels of genes related to trehalose biosynthesis or conidial viability in Δ*csgA* conidia. Further research on how CsgA regulates trehalose biosynthesis will be needed.

Δ*csgA* conidia exhibited a delayed germination rate and reduced germ tube length, indicating that CsgA regulates conidial germination in *A. nidulans*. In *S. cerevisiae*, spore germination is initiated by the sensing of nutrient sources, such as carbon sources, mediated by G-protein-coupled receptor proteins^38,39^. Notably, the G-protein signaling pathway-related gene, GanB, which positively regulates conidial germination by sensing carbon sources^34^, was downregulated in the Δ*csgA* strain, whereas RgsA, which downregulates GanB in response to carbon sources^28^, was upregulated. Taken together, these results suggest that CsgA plays a role in the regulatory mechanisms of conidial germination. Further research will be required to understand the specific mechanisms of *csgA* function in *A. nidulans* conidia.

*A. nidulans* produces sterigmatocystin as a secondary metabolite, which is a precursor to aflatoxin. It has been constantly reported that sterigmatocystin has been detected in green coffee beans, grains, and cheese. Because *A. nidulans* exists almost everywhere, controlling mycotoxin production is important for human health. We examined sterigmatocystin production in *A. nidulans* conidia and found that Δ*csgA* conidia showed increased sterigmatocystin production compared to those of the control and C’*csgA* strains. In addition, transcriptomic analyses showed an increase in mRNA expression of the sterigmatocystin cluster genes in the deletion strain (Fig. S3). Given these results, we conclude that CsgA negatively regulates sterigmatocystin production in *A. nidulans* conidia.

Similar to conidia, Δ*csgA* ascospores exhibited elevated trehalose content, improved stress tolerance, and increased sterigmatocystin production compared to control and C’*csgA* ascospores. As CsgA regulates the sexual development and germination of ascospores in *A. nidulans*, we infer that CsgA also plays a crucial role in ascospores. Further characterization of the role of CsgA in *A. nidulans* ascospores may reveal the overall role of CsgA in *A. nidulans* spores.

In summary, the roles of the conidia-specific TF CsgA were characterized in the model organism *A. nidulans*. Deletion of *csgA* resulted in increased conidiation, fungal growth, and abnormal sexual development in *A. nidulans*. Additionally, sterigmatocystin production increased in both conidia and ascospores in the Δ*csgA* strain. These results indicate that CsgA is essential for fungal development and mycotoxin production by *A. nidulans*.

In summary, the roles of the conidia-specific TF CsgA were characterized and demonstrated that CsgA is essential for appropriate fungal development, conidial maturation, and sterigmatocystin production in the model organism *A. nidulans.* Although, the role of CsgA was characterized in *A. nidulans*, the roles the homologs or orthologs of *csgA* have not been studied yet. Also, genetic role and network for CsgA have not been published yet. Therefore, further in-depth studies for the roles of CsgA orthologs in other fungal species and the downstream target genes and genetic network for CsgA will provide to understand fungal biology.

## Methods

### Strains, media, and culture conditions

The fungal strains used in the present study are listed in Table S1. Each strain was grown on solid or liquid minimal media (MM; pH 6.5; 5% nitrate salt solution composed of 120 g/L NaNO_3_, 10.4 g/L KCl, 10.4 g/L MgSO_4_∙H_2_O, 30.4 g/L KH_2_PO_4_, and 0.1% trace element solution pH 5.5 composed of 22 g/L ZnSO_4_∙7H_2_O, 11 g/L H_3_BO_3_, 5 g/L MnCl_2_∙4H_2_O, 5 g/L FeSO_4_∙7H_2_O, 1.6 g/L CoCl_2_∙5H_2_O, 1.6 g/L CuSO_4_∙5H_2_O, 1.1 g/L (NH_4_)_6_Mo_7_O_24_∙4H_2_O, and 50 g/L Na_2_EDTA)^40^ with 1% glucose (MMG) and incubated at 37 °C. Sexual media (SM; pH 6.5; 20 g/L glucose, 1.5 g/L glycine, 0.52 g/L MgSO_4_∙7H_2_O, 0.52 g/L KCl, 1.52 g/L KH_2_PO_4_, and 1 mL/L of 0.1% trace element solution)^41^ was used to induce sexual development in *A. nidulans*. To investigate the effects of *csgA* overexpression in *A. nidulans*, strains were inoculated on non-inducing solid MMG, inducing solid MMTY, or inducing solid YLC (pH 6.5; 0.1% yeast extract, 1.5% lactose, 30 mM cyclopentanone) at 37 °C for 5 days^21^. To confirm mycotoxin production, each strain was inoculated into liquid complete media (CM; pH 6.5; 20 g/L glucose, 5% nitrate salt solution, 0.1% trace element solution pH 5.5, 1.5 g/L casamino acids, and 2 g/L bacto-peptone) at 30 °C for 7 days^42^.

### Construction of the *csgA* deletion mutant strains

The oligonucleotides used to construct the deletion mutants are listed in Table S2. To generate deletion mutant strains of *A. nidulans*, gene deletion cassettes were constructed using double-joint PCR (DJ-PCR)^43^. First, the 5′- and 3′-flanking regions of *csgA* were amplified from the genomic DNA of *A. nidulans* FGSC4 (wild-type, WT), using the primer pairs DF/TailR (OHS1191/OHS1193) and TailF/DR (OHS1192/OHS1194). Next, the *Aspergillus fumigatus pyrG* marker (*AfupyrG*) was amplified from the genomic DNA of *A. fumigatus* AF293 (WT), using the primer pair 5′_*AfupyrG*_F/3′_*AfupyrG*_R (OHS1542/OHS1543). Finally, the three fragments, including the 5′- and 3′-flanking fragments and the *AfupyrG* marker, were combined and amplified using the primer pair NF/NR (OHS1195/OHS1196).

For transformation, conidia (1 × 10^8^ spores) from *A. nidulans* RJMP1.59 were inoculated in liquid yeast glucose medium (YG; pH 6.5; 2% glucose, 0.5% yeast extract, and 0.1% trace element solution) and incubated at 30 °C, 150 rpm for 13 h. Mycelia were harvested by filtering through sterile Miracloth (Calbiochem, San Diego, CA, USA) and cultured with VinoTastePro (Novozymes, Bagsvaerd, Denmark) to generate protoplasts^44^. Subsequently, deletion cassettes were introduced into the protoplasts. Transformants were inoculated onto solid MMG. The genomic DNA of transformants was isolated and deletions were confirmed by PCR, followed by restriction enzyme digestion. Three independent *csgA* deletion mutant strains were isolated.

### Construction of the *csgA* complemented and overexpressed strains

The plasmids used in the present study are listed in Table S3. To construct the *csgA* complemented strain, the *csgA* open reading frame (ORF) derived from *A. nidulans* FGSC4 genomic DNA and its predicted promoter region were amplified with the primer pair OHS1549/OHS1550. The PCR product was digested with NotI and cloned into pHS13^45^. The resulting plasmid, pHJ2.1, was introduced into Δ*csgA* strain THJ13.1, giving rise to THJ28.1. Three mutants were isolated and confirmed by PCR and qRT-PCR.

For *csgA-*overexpressing strains, a fusion construct under the *alcA* promoter was generated. The *csgA* ORF derived from *A. nidulans* FGSC4 genomic DNA was amplified using the primer pair OHS1733/OHS1550. The PCR product was digested with NotI and cloned into pHS3^46^, which contained the *A. nidulans alcA* promoter^45^.

The resulting plasmid, pHJ4.1, was then inserted into TNJ36^46^.

### Nucleic acid isolation and quantitative reverse-transcription PCR

Conidia (asexual spores) or mycelia (vegetative samples) were collected^47^. The samples were homogenized with zirconia/silica beads (RPI, Mt. Prospect, IL, USA) and TRIzol reagent (Invitrogen, Waltham, MA, USA) using a Mini-Bead beater (BioSpec Products Inc., Bartlesville, OK, USA)^48^. After homogenization, the samples were centrifuged and supernatants were transferred to new tubes and mixed with ice-cold isopropanol. After centrifugation, RNA pellets were washed with 70% ethanol and dissolved in RNase-free water. cDNA was synthesized using GoScript reverse transcriptase (Promega, Madison, WI, USA). qPCR was performed using iTaq Universal SYBR Green Supermix (Bio-Rad, Hercules, CA, USA) and CFX96 Touch Real-Time PCR (Bio-Rad). The β-*actin* gene was used as a control. All experiments were performed in triplicate.

### Ascospore germination analysis

Each strain was point-inoculated onto solid SM and incubated at 37 °C for 7 and 14 days. After incubation, ten size-matched cleistothecia were isolated from the plates and washed with ddH_2_O with 0.01% Triton X-100 (Sigma-Aldrich, St Louis, MO, USA)^49^. Each cleistothecium was transferred to a new tube and crushed to collect ascospores. The number of ascospores was counted using a hemocytometer. After a series of dilutions, 100 ascospores were spread onto solid MMG and incubated at 37 °C for 2 days. The number of colonies was counted and survival rates were calculated as the ratio of the number of viable colonies to the number of inoculated ascospores.

### Trehalose analysis

Two-day-old conidia (2 × 10^8^ spores) were collected using ddH_2_O with 0.01% Triton X-100 (Sigma-Aldrich)^50^. The samples were centrifuged, resuspended in 200 μL of fresh ddH_2_O with 0.01% Triton X-100, and incubated in a 95 °C heat block for 20 min. After incubation, the samples were centrifuged and 100 μL of the supernatant was transferred to new tubes. The supernatants were mixed with 100 μL of 0.2 M sodium citrate (pH 5.5) and incubated with or without trehalase (3 mU, Sigma-Aldrich) at 37 °C for 8 h. All experiments were performed in triplicate.

### Spore viability assay

Two- and ten-day-old conidia were collected from each strain using ddH_2_O with 0.01% Triton X-100 (Sigma-Aldrich)^50^. The number of conidia was counted using a hemocytometer. Approximately 100 conidia were spread on solid MMG and incubated at 37 °C for 2 days in dark conditions. After incubation, colonies were counted and survival rates were calculated as the ratio of the number of viable colonies to the number of spores inoculated.

### Stress tolerance tests

To evaluate thermal stress tolerance, 2-day-old conidia (1 × 10^3^ per mL) were incubated for 30 min in a 55 °C heat block^51^. After incubation, 100 conidia were spread on solid MMG and incubated at 37 °C for 2 days in dark conditions. Colonies were counted and survival rates were calculated as the ratio of the number of grown colonies relative to the number of conidia not treated with heat.

To evaluate UV stress tolerance, approximately 100 conidia were spread on solid MMG^51^. The plates were placed in a UV crosslinker (Spectrolinke XL-1000 UV crosslinker; Thomas Scientific, Swedesboro, NJ, USA). UV light was irradiated on the plates, which were subsequently incubated at 37 °C for 2 days in dark conditions. After incubation, colonies were counted and survival rates were calculated.

To evaluate oxidative stress tolerance, two-day-old conidia (1 × 10^3^ per mL) with 0.1 M of H_2_O_2_ were incubated at 37 °C for 30 min^51^. After incubation, 100 conidia were spread onto solid MMG and incubated at 37 °C for 2 days in dark conditions. Colony numbers were counted and survival rates were calculated. All stress tolerance tests were performed in triplicate.

### RNA-sequencing analysis

Two-day-old conidia from WT and Δ*csgA* strains were collected using 0.01% Triton X-100 (Sigma-Aldrich)^50^. RNA isolation was performed using the TRIzol (Invitrogen) method described above^49^. The prepared RNA samples were sent to Theragen Bio (Seongnam, South Korea) and quality control, cDNA library construction, and sequencing were successively performed. A cDNA library was constructed using the TruSeq Stranded mRNA Sample Prep Kit (Illumina, San Diego, CA, USA). The quality of the constructed library was evaluated using an Agilent High-Sensitivity DNA Kit (Agilent Technologies, Santa Clara, CA, USA). Sequencing was performed on an Illumina HiSeq2500 sequencer (Illumina).

RNA-Seq data analysis was performed as previously described^50^. Low-quality reads were excluded, and filtered reads were mapped to the *A. nidulans* A4 transcriptome using the STAR v.2.3.0e aligner^52^. Gene expression levels were examined with Cufflinks v2.2.1^53^ using the gene annotation reference provided by *Aspergillus* Genome Database (AspGD)^54^. To perform differential expression gene (DEG) analysis, gene-level counts data were generated using the HTSeq-count v0.11.2 with the options “-m intersection-nonempty” and “-r option considering paired-end sequence”^55^. DEGs were identified using TCC v1.26.0^56^. The normalization factors were calculated using the iterative DEGES/edgeR method. DEGs were sorted based on a q-value threshold of < 0.05. All RNA-seq data files were obtained from the NCBI BioProject database (PRJNA800619).

### GO enrichment analysis

GO enrichment analyses were performed using the database provided by AspGD^54^ and the GO analysis tool provided by FungiFun^27^. To analyze the genes identified by DEG analysis, a GO-based trend test was performed using Fisher’s exact test, and p-values < 0.001 were considered statistically significant.

### Conidial germination analysis

Two-day-old conidia from each strain were collected^57^. Approximately 10^7^ conidia were spread onto solid MM, with or without glucose, and incubated at 37 °C. Germ tube length and germination rate were measured every hour until the germination rate reached 100%. The measurements were conducted using a Zeiss Lab.A1 microscope equipped with AxioCam 105c and AxioVision (Rel. 4.9) Digital Imaging Software.

### Mycotoxin extraction and thin-layer chromatography

To extract mycotoxins from *A. nidulans*, approximately 10^7^ conidia were inoculated into 5 mL of liquid CM and incubated at 30 °C for 7 days in dark conditions^21^. After incubation, CHCl_3_ was added to the culture medium and vortexed to mix evenly. After centrifugation, the separated organic phases were transferred to new vials. The samples were absolutely evaporated in a 60 °C oven, and 50–100 μL of CHCl_3_ was added for resuspension. The samples were spotted onto a TLC silica plate (Kieselgel 60, 0.25 mm; Merck KGaA, Darmstadt, Germany) and placed in a chamber containing a solution of toluene, ethyl acetate, and acetic acid (8:1:1, v/v/v). The TLC plate was treated with 1% ammonium hydroxide hydrate (Sigma-Aldrich). Images of TLC plates were captured under UV light (366 nm). Quantification of sterigmatocystin band intensities was performed using ImageJ software. Experiments were performed in triplicates for each strain.

### Microscopy

Photographs of the colonies were taken using a Pentax MX-1 digital camera. The morphology of the colonies was investigated using a Zeiss Lab.A1 microscope equipped with an AxioCam 105c and AxioVision (Rel. 4.9) Digital Imaging Software.

### Statistical analysis

GraphPad Prism version 5.01 software was used for statistical analyses. Statistical differences between control and mutant strains were evaluated using Student’s unpaired *t* tests. Data are reported as the mean ± standard deviation. Statistical significance was set at p < 0.05.

## Supplementary Information

Supplementary Information.

## Data Availability

All RNA-seq data files were obtained from the NCBI BioProject database (PRJNA800619). Other data are available upon request from the corresponding author.
