# Supplementary material for: The function of a conidia specific transcription factor CsgA in Aspergillus nidulans
Source: Sci Rep. 2022 Sep 16;12:15588. doi: 10.1038/s41598-022-19749-6 (PMC9481610; doi:10.1038/s41598-022-19749-6)
Supplement: Supplementary file 1 — Supplementary Information. [file 41598_2022_19749_MOESM1_ESM.pdf]

Supplementary material

The function of a conidia specific transcription factor CsgA in *Aspergillus nidulans*

Short title: The roles of CsgA in *A. nidulans*

He-Jin Cho[1] and Hee-Soo Park[1,2*]

[1] School of Food Science and Biotechnology, Kyungpook National University, Daegu, 41566, Republic of Korea

[2] Department of Integrative Biology, Kyungpook National University, Daegu, 41566, Republic of Korea

† Corresponding Author:
Hee-Soo Park
School of Food Science and Biotechnology, Kyungpook National University
80 Daehak-ro, Buk-gu, Daegu, 41566, Republic of Korea
Tel: +82-53-950-5751;
Fax: +82-53-950-6750
Email: phsoo97@knu.ac.kr

A

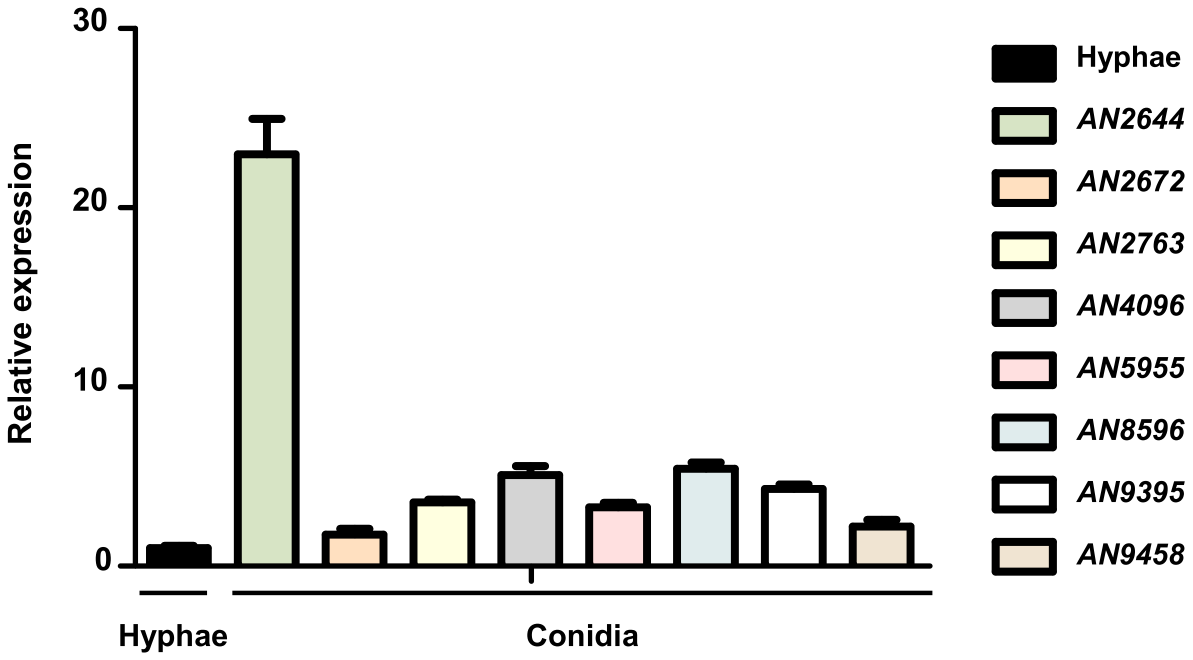

B

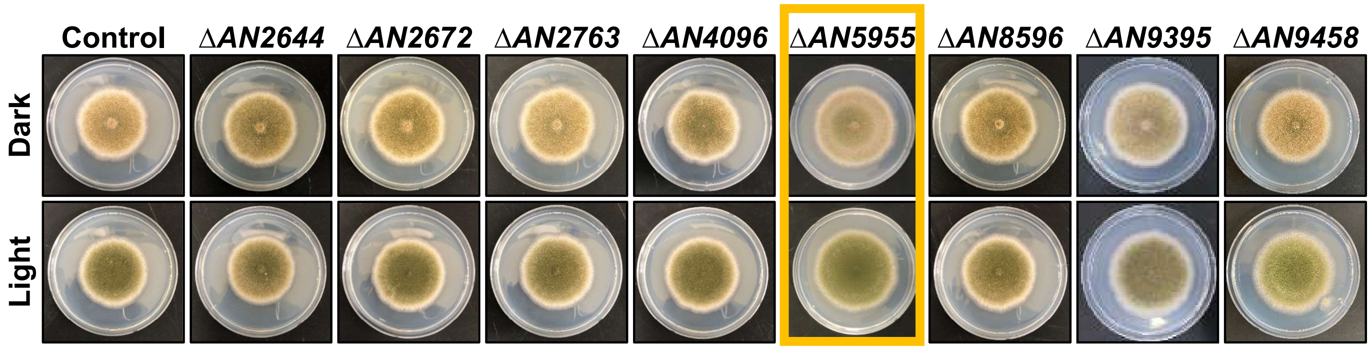

Supplementary Figure. S1. Identification of CsgA (A) Quantitative analyses of relative expression levels of 8 zinc cluster TFs in conidia compared to hyphae. (B) Colony photographs of control and deletion mutants of zinc cluster TFs.

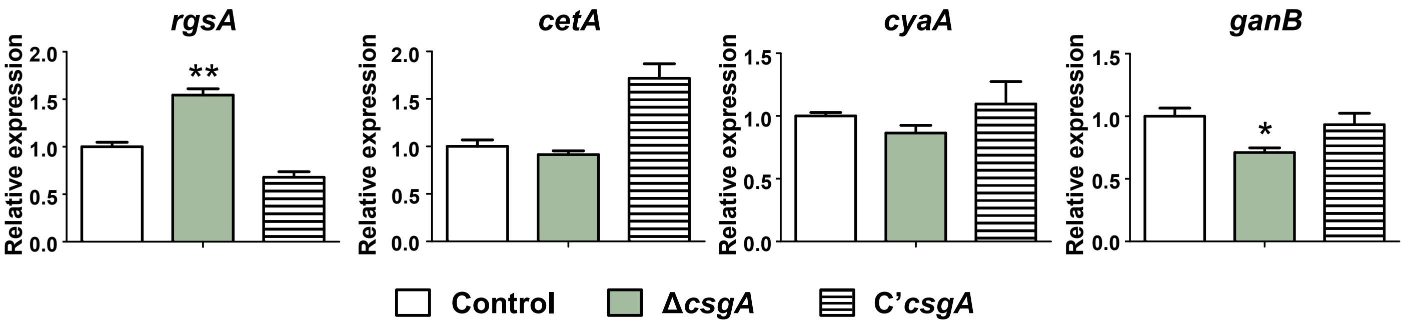

Supplementary Figure. S2. The role of CsgA in conidial germination qRT-PCR analyses of germination related genes that were down-regulated in the Δ*csgA* strain.

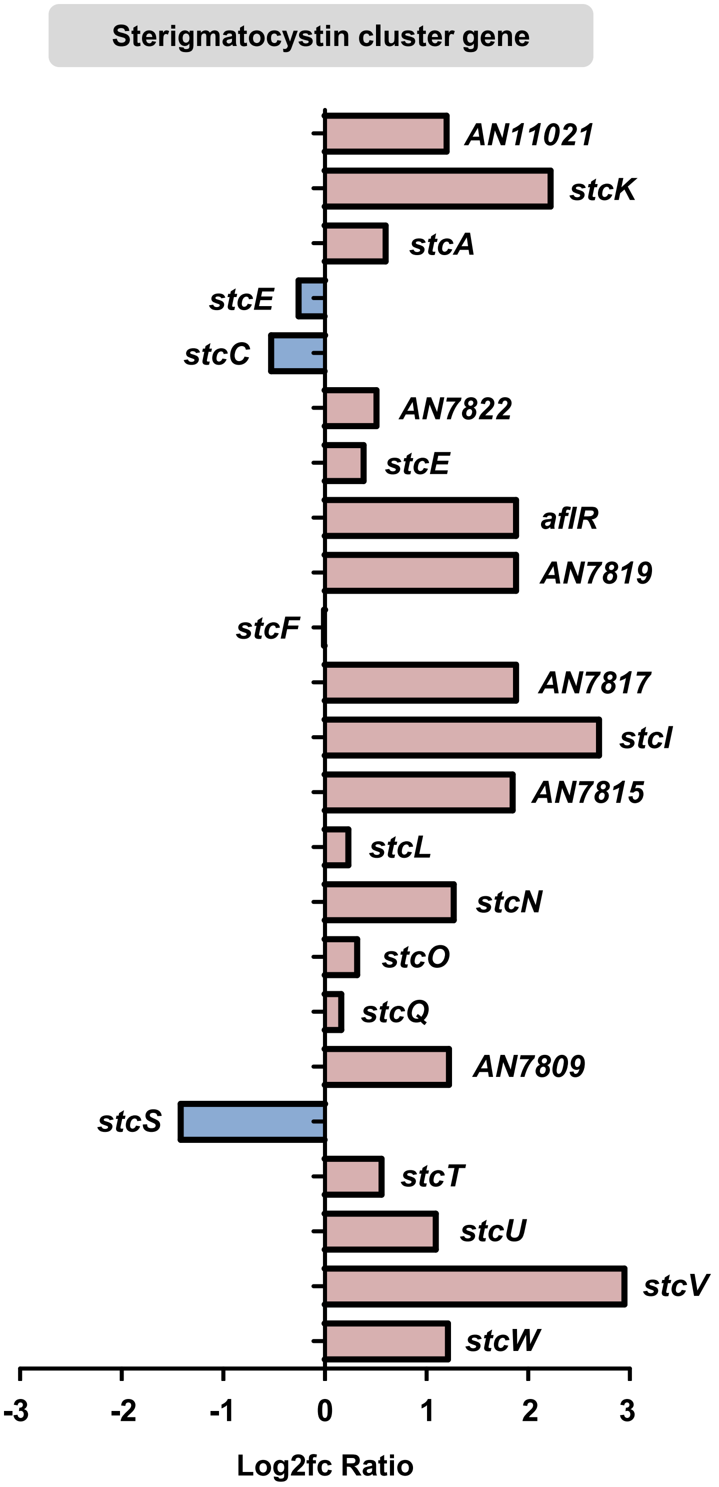

Supplementary Figure. S3. The mRNA expression of sterigmatocystin cluster gene in *csgA* deletion mutant strains. The mRNA expression levels of genes participated in sterigmatocystin production in the Δ*csgA* conidia.

7-days-old conidia

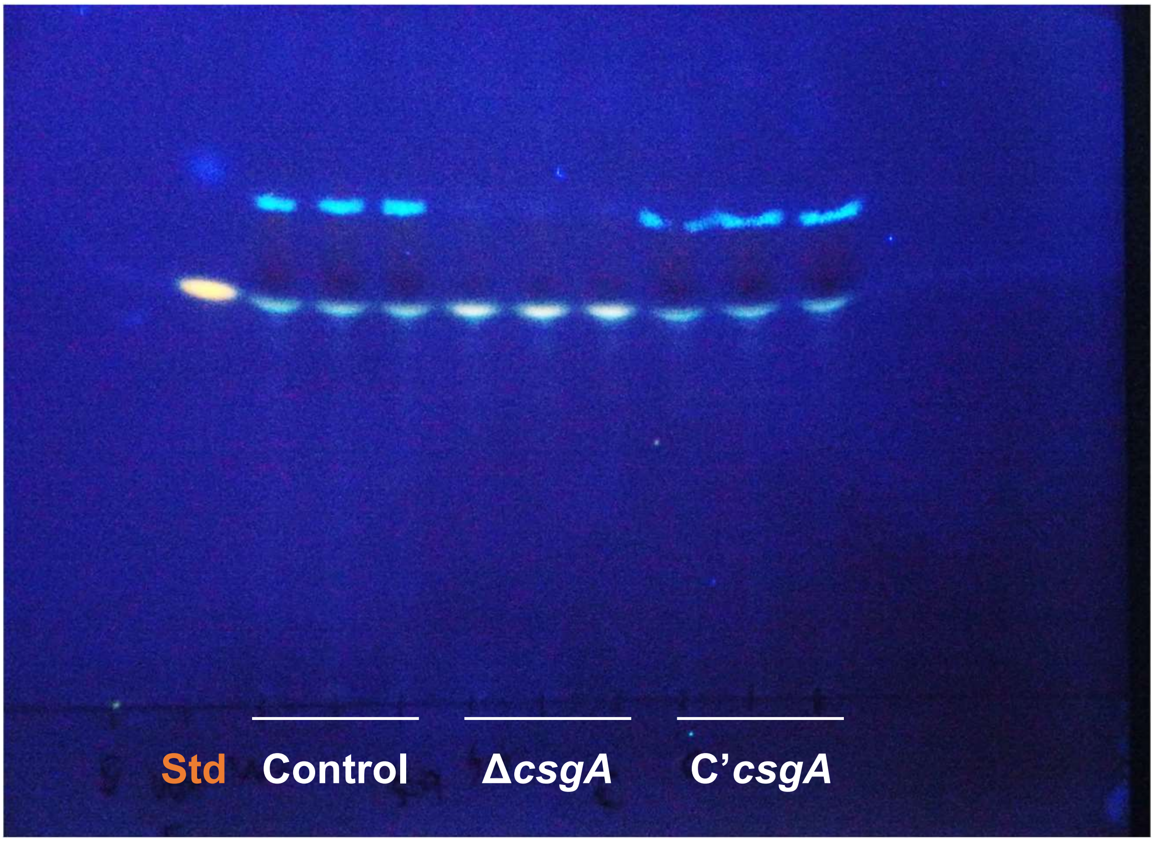

*** Std: Sterigmatocystin**

Supplementary Figure. S4. Image for whole TLC plate showing the production of sterigmatocystin by 7-day-old conidia from control (TNJ36), Δ*csgA* (THJ13.1), and C'*csgA* (THJ28.1) strains shown in **Figure 8A**.

14-days-old ascospore

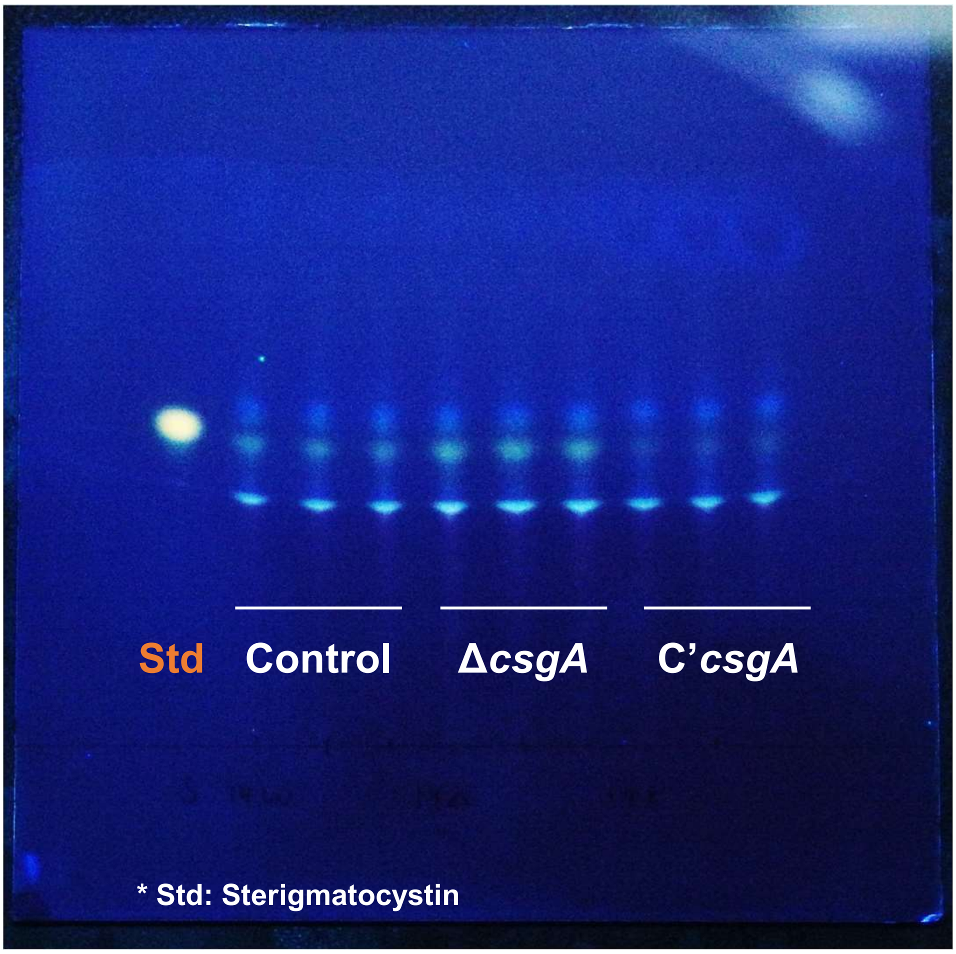

Supplementary Figure. S5. Image for whole TLC plate showing the production of sterigmatocystin by 14-day-old ascospores from control (TNJ36), Δ*csgA* (THJ13.1), and C'*csgA* (THJ28.1) strains shown in **Figure 9D**.

Supplementary Table S1: Strains used in this study.

Strain	Relevant genotype	References
FGSC4	*A. nidulans* Wild type, *veA*$^+$	FGSC[a]
TNJ36	*pyrG89*; *pyroA4; pyrG*$^+$*,veA*$^+$	[41]
RJMP1.59	*pyrG89*; *pyroA4, veA*$^+$	[53]
THS30.1	*pyrG89 AfupyrG*$^+$*, veA*$^+$	[54]
THJ13.1~3	*pyrG89*; *pyroA4*; Δ*csgA*::*AfupyrG*$^+$; *veA*$^+$	This study
THJ28.1~2	*pyrG89*; *pyroA*::*csgA*(*p*)::*csgA*::FLAG$_{3x}$::*pyroA*[b]; Δ*csgA*::*AfupyrG*$^+$; *veA*$^+$	This study
THJ27.1~2	*pyrG89*; *AfupyrG*$^+$; *pyroA*::*alc(p)*::*csgA*::*FLAG*:: *pyroA*[b]*; veA*$^+$	This study

[a]Fungal Genetic Stock Center

[b]The 3/4 *pyroA* marker causes targeted integration at the *pyroA* locus

Supplementary Table S2: Oligonucleotides used in this study.

Name	Sequence (5'→3')[a]	Purpose
**OHS1542**	CCTGGTCTTTGGTTTGGTACACC	5' *AfupyrG* marker_F
**OHS1543**	CGACTGGCAGGAGATGATCC	3' *AfupyrG* marker_R
**OHS1191**	AAGGCCCACCGGACTAACA	5' *csgA* DF
**OHS1192**	GCCATCGTCGAAGTTGACC	3' *csgA* DR
**OHS1193**	*GGCTTTGGCCTGTATCATGACTTCA* TTCGGCAGTTCAGGTACGG	3' *csgA* with *AfupyrG* tail
**OHS1194**	*TTTGGTGACGACAATACCTCCCGAC* CGAACGTGGATATCCTAGCG	5' *csgA* with *AfupyrG* tail
**OHS1195**	CTGATAGGCCTGGATTGTCG	5' *csgA* NF
**OHS1196**	GCGTACAACCATGTCAGCC	3' *csgA* NR
**OHS1197**	TCACGTCTCCAAGCTTTCCT	5' *csgA* RT_F
**OHS1198**	TCTCGAAGTCTTGCTGCTCT	3' *csgA* RT_R
**OHS1549**	*aatt* **GCGGCCGC** CCGCTCCACACAACCAGA	5' *csgA Not*I_F
**OHS1550**	*aatt* **GCGGCCGC** GCGAGCAACTGAAACACG	3' *csgA Not*I_R
**OHS1733**	*aatt* **GCGGCCGC** ATGGCCTCCAACAGCAAA	5' *csgA* OE_F with *Not*I
**OHS0044**	GTAAGGATCTGTACGGCAAC	5' *actin* RT_F
**OHS0045**	AGATCCACATCTGTTGGAAG	3' *actin* RT_R
**OHS0580**	CAAGGCATGCATCAGTACCC	5' *brlA* RT_F
**OHS0581**	AGACATCGAACTCGGGACTC	3' *brlA* RT_R
**OHS0777**	GGGAGCGAACAGTCTCACTA	5' *mutA* RT_F
**OHS0778**	GTCGATTCCCGTTTCCTTGG	3' *mutA* RT_R
**OHS0576**	GGTTGAAGTCGTCGGTTGAG	5' *tpsA* RT_F
**OHS0577**	TGGAAACCGATGAGGTCACA	3' *tpsA* RT_R
**OHS0616**	CTCCTACTCGCGTCACTTCT	5' *orlA* RT_F
**OHS0617**	AGGAAAGACATCCACAGCCA	3' *orlA* RT_R

[a]Tail sequences are shown in italics. Restriction enzyme sites are in bold.

Supplementary Table S3: Oligonucleotides used in this study.

Plasmid	Relevant genotype	References
pHS13	*FLAG*$_{3x}$::*trpC*(*t*)::3/4*pyroA*	[40]
pHJ2.1	*csgA*(*p*)::*csgA*::*FLAG*$_{3x}$::*trpC*(*t*)::3/4*pyroA*	This study
pHS3	*alc*(*p*)::*FLAG*$_{3x}$::*trpC*(*t*)::3/4*pyorA*	[41]
pHJ4.1	*alc*(*p*)::*csgA*::*FLAG*$_{3x}$::*trpC*(*t*)::3/4*pyroA*	This study
